# Increased Transendothelial Transport of CCL3 Is Insufficient to Drive Immune Cell Transmigration through the Blood–Brain Barrier under Inflammatory Conditions In Vitro

**DOI:** 10.1155/2017/6752756

**Published:** 2017-05-25

**Authors:** Maxime De Laere, Carmelita Sousa, Megha Meena, Roeland Buckinx, Jean-Pierre Timmermans, Zwi Berneman, Nathalie Cools

**Affiliations:** ^1^Laboratory of Experimental Hematology, Vaccine & Infectious Disease Institute (VAXINFECTIO), Faculty of Medicine and Health Sciences, University of Antwerp, 2610 Wilrijk, Belgium; ^2^Laboratory of Cell Biology and Histology, Faculty of Pharmaceutical, Biomedical and Veterinary Sciences, University of Antwerp, 2610 Wilrijk, Belgium; ^3^Center for Cell Therapy and Regenerative Medicine, Antwerp University Hospital, 2650 Edegem, Belgium

## Abstract

Many neuroinflammatory diseases are characterized by massive immune cell infiltration into the central nervous system. Identifying the underlying mechanisms could aid in the development of therapeutic strategies specifically interfering with inflammatory cell trafficking. To achieve this, we implemented and validated a blood–brain barrier (BBB) model to study chemokine secretion, chemokine transport, and leukocyte trafficking in vitro. In a coculture model consisting of a human cerebral microvascular endothelial cell line and human astrocytes, proinflammatory stimulation downregulated the expression of tight junction proteins, while the expression of adhesion molecules and chemokines was upregulated. Moreover, chemokine transport across BBB cocultures was upregulated, as evidenced by a significantly increased concentration of the inflammatory chemokine CCL3 at the luminal side following proinflammatory stimulation. CCL3 transport occurred independently of the chemokine receptors CCR1 and CCR5, albeit that migrated cells displayed increased expression of CCR1 and CCR5. However, overall leukocyte transmigration was reduced in inflammatory conditions, although higher numbers of leukocytes adhered to activated endothelial cells. Altogether, our findings demonstrate that prominent barrier activation following proinflammatory stimulation is insufficient to drive immune cell recruitment, suggesting that additional traffic cues are crucial to mediate the increased immune cell infiltration seen in vivo during neuroinflammation.

## 1. Introduction

Until recently, the central nervous system (CNS) was considered to be an immunologically isolated and inert compartment. Given the presence of the blood–brain barrier (BBB) on the one hand and the apparent lack of a draining lymphatic system on the other hand, the CNS appeared to be anatomically separated from the peripheral immune system [1, 2]. However, the concept of this so-called immune privilege has been drastically reviewed and we now know that the CNS is an immune-competent and particularly immune-specialized compartment. In this context, a better understanding of the mechanisms of leukocyte trafficking into the CNS might yield insights into the regulation of protective or pathologic immune responses in the brain. Although the general paradigm of leukocyte trafficking also applies to immune cell recruitment to the CNS, the specialized structure of the BBB critically modulates this process [3–5]. Its distinguished architecture comprising tight junctions and its low basal expression of adhesion molecules ensure a limited but steady entry of immune cells in normal physiological conditions, while its activation and breakdown during neuroinflammation are associated with massive infiltration of circulating leukocytes into the CNS. In both situations, chemokines secreted by and displayed on the luminal surface of endothelial cells of the BBB play a crucial role in regulating and directing immune cell entry into the CNS.

Chemokines are low molecular weight chemotactic proteins belonging to the cytokine family. Although in the CNS, several chemokines have been shown to be involved in neuronal development and survival as well as in synaptic transmission [6], their generic function is to induce cell migration and regulate immune cell trafficking to and within the CNS. In this perspective, chemokines presented at the CNS barriers, and in particular at the BBB, are crucial in the recruitment of circulating immune cells to the CNS. These chemokines are presented to passing leukocytes following production by cells of the BBB or following secretion by CNS-resident or infiltrating cells and subsequent transport to the endothelial cell surface.

Primary human brain microvascular endothelial cells (hBMEC) are known to constitutively express minimal levels of CXCL8 [7], CXCL10 [8], CXCL12 [9], CCL2 [10, 11], CCL3 [11], CCL4 [12], and CCL5 [12] and to strongly upregulate the expression of these chemokines upon activation by CD40 ligation [10], stimulation with proinflammatory cytokines [7, 10–12], or viral infection [8]. While the human cerebral microvascular endothelial cell line hCMEC/D3 represents a simple and well-accepted tool and a valid alternative to primary hBMEC to study BBB characteristics and leukocyte transmigration in vitro [13], the pattern of chemokine secretion of the hCMEC/D3 cell line has not been studied as thoroughly.

In addition to chemokine production at the BBB, major sources of inflammatory chemokines can also be found behind the BBB, that is, within the CNS itself. More specifically, involvement of CNS-derived CCL3 in neuroinflammation has been demonstrated and its sources have been identified. Expression of CCL3 is upregulated in the CNS during multiple sclerosis (MS) [14, 15] and its experimental model experimental autoimmune encephalomyelitis [16–18], Alzheimer's disease [19, 20], seizure disorders [21–25], and viral [26, 27] as well as bacterial neuroinflammation [28, 29] and is mainly localized in microglia and perivascular macrophages [15, 30]. Also, human primary astrocytes have been shown to produce CCL3 in vitro after proinflammatory stimulation, suggesting that they might be an important source of CCL3, among other chemokines, during neuroinflammation as well [31]. However, to stimulate recruitment of circulating leukocytes to the site of inflammation, the chemokines produced abluminally—including CCL3—need to cross the BBB to reach the vascular lumen. Since paracellular diffusion is unlikely given the highly restrictive nature of the BBB, other mechanisms are thought to be involved in this process. For instance, transport of CCL2 across an in vitro model consisting of a monolayer of murine brain microvascular endothelial cells was shown to occur mainly via transcytosis, a process which also involved C-C-chemokine receptor 2 (CCR2) [32]. Once secreted into the vascular lumen or presented on the endothelial cell surface, chemokines orchestrate the recruitment of immune cells to the CNS. In this study, we aim to ultimately expand our current knowledge of the mechanisms underlying leukocyte transmigration under steady-state and inflammatory conditions, by studying chemokine secretion and transport in an in vitro BBB model consisting of the hCMEC/D3 cell line and primary human astrocytes.

## 2. Materials and Methods

### 2.1. Cell Culture

The hCMEC/D3 cell line (Tébu-Bio, Le Perray-en-Yvelines, France) was kept in the exponential growth phase in microvascular endothelial cell growth (EGM-2-MV) medium (Lonza, Verviers, Belgium) consisting of endothelial cell basal (EBM-2) medium supplemented with hydrocortisone, ascorbic acid, vascular endothelial growth factor (VEGF), human basic fibroblast growth factor (bFGF), recombinant human insulin-like growth factor-1 (R^3^-IGF-1), human epidermal growth factor (EGF), gentamicin, amphotericin-B, and 2.5% fetal calf serum (FCS), as recommended by the manufacturer in flasks coated with type I rat tail collagen (Sigma; Diegem, Belgium). Human primary astrocytes (Sanbio, Uden, The Netherlands) were grown in poly-L-lysine-coated flasks in astrocyte medium (Sanbio), according to the manufacturer's instructions. Cells were maintained in a humidified atmosphere supplemented with 5% CO_2_ at 37°C.

BBB cultures were established by coculturing astrocytes (passages 1–3) and hCMEC/D3 endothelial cells (passages 30–33) on opposing sides of a 24-well cell culture insert with 3.0 *μ*m pores (Greiner Bio-one, Vilvoorde, Belgium). For this, the inserts were coated with poly-L-lysine on the underside and type I collagen on the topside. Astrocytes were seeded at a density of 15,000 cells per cm^2^ on the insert underside and were allowed to adhere for 2 hours, while replenishing the medium every 10 to 15 minutes. Subsequently, inserts were transferred into a medium-filled well and hCMEC/D3 endothelial cells were seeded onto the insert upper side at a density of 25,000 cells per cm^2^. Cultures were maintained in EGM-2 MV medium in 5% CO_2_ at 37°C. Three days after initiating the coculture, the growth medium was replaced by EBM-2-plus medium, consisting of EBM-2 medium supplemented with 1.4 *μ*M hydrocortisone, 1 ng/ml bFGF, 10 *μ*g/ml gentamicin, 1 *μ*g/ml amphotericin-B, and 2.5% FCS. EBM-2-plus medium was replenished every other day. Functional assays were performed between days 10 and 13 of culture. One day before the assay, EBM-2-plus medium was replaced by serum-reduced EBM-2-plus medium, that is, supplemented with 0.25% FCS. When indicated, the cocultures were stimulated with 50 U/ml TNF-*α* and/or 50 U/ml IFN-*γ* for 16 to 18 hours, while hydrocortisone was omitted from the medium [33].

### 2.2. TEER Measurement

Transendothelial electrical resistance (TEER) was determined using the EVOM-2 voltohmmeter with STX electrodes (World Precision Instruments, Hitchin, Hertfordshire, United Kingdom). Measurements were performed in duplicate and the final TEER value, expressed in Ωcm^2^, was obtained by subtracting background TEER values, that is, mean TEER across an empty insert, from the mean TEER value recorded across hCMEC/D3 monolayers or BBB cocultures.

### 2.3. FITC-Dextran Permeability Assay

For assessing permeability of BBB cocultures to the tracer molecule FITC-dextran, 100 *μ*l of a 100 *μ*g/ml 4 kDa FITC-dextran solution was added to the upper compartment and fluorescence recovery in the lower chamber was measured after 60, 120, and 180 minutes using a Victor^3^ multilabel fluorometer. As a positive control, 100 *μ*l of 100 *μ*g/ml FITC dextran was directly added into the lower chamber, yielding a final concentration of 14.29 *μ*g/ml. The negative control consisted of medium only. The percentage fluorescence recovery was calculated as follows: [fluorescence_(lower compartment)_ − fluorescence_(negative control)_]/[fluorescence_(positive control)_ − fluorescence_(negative control)_] ∗ 100%.

### 2.4. RNA Isolation and Quantitative Real-Time Polymerase Chain Reaction (qPCR)

For analysis of tight junction and adhesion molecule expression, total RNA from hCMEC/D3 endothelial cells, which were cocultured with astrocytes in an in vitro BBB model, was isolated. For this, cells were disrupted and homogenized with guanidine-thiocyanate-containing lysis buffer. Before cell lysis, astrocytes were detached enzymatically and removed mechanically from the insert underside. As a control, hCMEC/D3 monolayer cultures, that is, without cocultured astrocytes, were used. Total RNA was isolated using the RNeasy microkit (Qiagen, Antwerp, Belgium). The RNA concentration was determined by measuring absorbance at 260 nm using a Nanodrop spectrophotometer (Wilmington, DE, USA). Reverse transcription of the obtained RNA into cDNA was performed using the iScript™ Advanced cDNA Synthesis Kit (Bio-Rad, Temse, Belgium). Subsequently, the SYBR® Green technology was used for relative mRNA quantification by qPCR in a CFX96 C1000 thermal cycler (Bio-Rad). qPCR reactions were conducted at 95°C for 2 minutes, followed by 40 cycles at 95°C for 5 seconds and at 60°C for 30 seconds. All primer sets were obtained from Bio-Rad; validation data can be found in Supplementary Table 1 available online at https://dx.doi.org/10.1155/2017/6752756. qPCR was performed in triplicate and resulting mRNA levels were normalized to levels of the reference gene actin-beta (ACTB). Melt curve analysis was performed to confirm the specificity of the amplified product. Bio-Rad CFX manager v3.1 was used for data processing and analysis.

### 2.5. Chemokine Secretion Assay

hCMEC/D3 endothelial cells and astrocytes cocultured or monocultured on cell culture inserts were pretreated with TNF-*α* and/or IFN-*γ* as described above. After 18 hours of stimulation, the proinflammatory cytokines were removed by extensive washing and fresh medium was added to the inserts. After 24 hours, culture supernatant was collected and stored at −20°C until analysis of chemokine concentration. Unstimulated cultures were used as control. Secreted chemokine levels were measured by U-PLEX assay (CCL2, CCL3, CCL4, CCL19, CXCL8, CXCL10, CXCL12, and CX_3_CL1) or MSD standard assay kit (CCL5) (Meso Scale Discovery, Rockville, MD, USA) according to the manufacturer's instructions.

### 2.6. Migration Assay

Peripheral blood mononuclear cells (PBMC) were isolated by density gradient centrifugation (Ficoll Pacque PLUS, GE Healthcare, Amsterdam, The Netherlands). Transmigration of PBMC was studied across steady-state and cytokine-activated BBB cocultures. To this end, EBM-2-plus medium was replaced by serum-reduced EBM-2-plus medium the day before the migration assay and cocultures were stimulated with 50 U/ml TNF-*α* and 50 U/ml IFN-*γ* for 16 to 18 hours, when indicated. After activation, BBB cocultures were washed twice with Iscove's Modified Dulbecco's Medium (IMDM) (Thermo Fischer Scientific) supplemented with 1% human AB serum (hAB) (Thermo Fischer Scientific) to remove proinflammatory stimuli. Subsequently, 5 × 10^5^ PBMC resuspended in IMDM supplemented with 1% human AB serum were added to the upper compartment. As a positive control, 5 × 10^5^ PBMC were added directly to the lower compartment. After 20 hours, migrated cells were collected from the basolateral compartment, while nonmigrating cells were recovered from the upper compartment. Recovered cells were counted using a Cyflow ML flow cytometer (Partec, Münster, Germany). For this, events were recorded at a fixed flow rate during 200 seconds. Simultaneously, flow cytometric analysis of chemokine receptor expression by PBMC was done using anti-CCR5-phycoerythrin-cyanine-7 (BD Biosciences), anti-CCR1-phycoerythrin-vio770 (Miltenyi Biotec, Leiden, The Netherlands), and anti-CCR4-phycoerythrin-CF594 (BD Biosciences) antibodies and isotype-matched controls. Results were analyzed using Flowjo software (Tree Star, Ashland, OR, USA). The percentage migration was calculated as follows: [event count_(migrated  cells)_/event count_(positive  control)_]∗100%.

### 2.7. Immunofluorescence Imaging

In some experiments, BBB cocultures were recovered for immunofluorescence imaging after performing the migration assay. For this, the BBB cocultures were fixed, blocked, and permeabilized using 4% paraformaldehyde (Sigma), and 0.01 M PBS (pH 7.4) supplemented with 0.05% thimerosal, 10% normal horse serum, and 1% triton X-100, respectively. Subsequently, the cells were stained overnight using the following primary antibodies: a mouse anti-CD45 antibody (1/100) (BD) and a rabbit anti-CD31 antibody (1/50) (Abcam, Cambridge, United Kingdom). Next, cell cultures were stained with a secondary FITC-labeled donkey anti-mouse antibody (1/100) and a secondary Cy3-labeled donkey anti-rabbit antibody (1/200) (Jackson ImmunoResearch, Newmarket, United Kingdom) for 2 hours at room temperature. Finally, cells were counterstained with 4′,6-diamidino-2-phenylindole (DAPI) (Sigma), and the transwell membrane containing the cells was mounted in citifluor (Citifluor Ltd., London, United Kingdom) following careful removal from the transwell insert and subsequently stored at 4°C. Confocal fluorescence microscopy analysis was performed for 3 donors. Per transwell membrane, 12 images were obtained using the UltraVIEW Vox spinning disk confocal system (Perkin Elmer, Waltham, Massachusetts, USA). Counting of CD45^+^ cells was performed manually using the ImageJ software (National Institutes of Health, Bethesda, Maryland, USA).

### 2.8. Chemokine Transport Assay

Basolateral-to-apical transport of CCL3 across the in vitro BBB model under steady-state and inflammatory conditions was assessed. Activation of the BBB cocultures was obtained by pretreating the cultures with TNF-*α* and IFN-*γ* for 16–18 hours, as described above. In some experiments, the involvement of the chemokine receptors CCR1 and CCR5 was studied using respective blocking antibodies (10 *μ*g/ml) (R&D, Abingdon, UK). For this purpose, cocultures were preincubated with the antibody for 1 hour at 37°C. An isotype-matched antibody (R&D) was used as a control. After washing, BBB cocultures were transferred into a new well containing serum-reduced EBM-2-plus medium supplemented with 10 ng/ml CCL3 (R&D) to study chemokine transport. As a control, serum-reduced EBM-2-plus medium without CCL3 was used to quantify secretion of endogenous CCL3. To discriminate between active transendothelial transport and passive paracellular leakage of exogenously added CCL3, experiments were performed at 37°C and at 4°C, respectively. After 60 minutes, the medium from the apical side was collected. Supernatant was stored at −20°C until analysis of CCL3 concentration by means of ELISA (eBioscience, Vienna, Austria), according to the manufacturer's instructions.

### 2.9. Statistical Analysis

qPCR data were analyzed using the CFX Manager software version 3.1 (Bio-Rad). All other data were analyzed using the GraphPad Prism software version 5.01 (GraphPad, San Diego, CA, USA). For the comparison of 2 groups, an unpaired Student's *t*-test was used. When comparing 3 groups or more, statistical analysis was performed by one-way ANOVA, followed by Tukey's multiple comparisons test or by a Kruskal–Wallis test in combination with Dunn's multiple comparison test in case data were not normally distributed according to a D'Agostino and Pearson omnibus normality test. Where applicable, results were analyzed by two-way ANOVA with a Bonferroni post hoc test. For qPCR results, differences were considered significant when *p* < 0.01. For all other data, statistical significance was considered when *p* < 0.05. Data are shown as mean ± standard error of the mean (SEM).

## 3. Results

### 3.1. The Functional Barrier Formed by an In Vitro Model of the BBB Demonstrates Reduced Barrier Integrity but Enhanced Expression of Adhesion Molecules ICAM-1 and VCAM-1, Following Proinflammatory Stimulation

The in vitro BBB model was characterized using RT-qPCR and assays measuring barrier function (Supplementary Table 1 and Supplementary Figure 1). Following start of coculture, TEER values gradually increased and from day 10 onwards, the level of TEER was significantly higher as compared to the initial value registered on day 3 (30.63 ± 1.11 Ωcm^2^ versus 18.19 ± 1.18 Ωcm^2^, *p* < 0.001, Supplementary Figure 1A). Accordingly, subsequent functional assays were performed between days 10 and 13 after initiation of the coculture. Astrocytes had only limited effects on the barrier function, as no differences in the gene expression profile of hCMEC/D3 cells following coculture with astrocytes could be detected, except for mRNA encoding occludin which was significantly upregulated as compared to monocultured hCMEC/D3 (Supplementary Figure 2B). However, the increased expression of this tight junction molecule was not mirrored by an increase in TEER in BBB cocultures when compared to hCMEC/D3 monocultures (Supplementary Figure 1C).

Proinflammatory stimulation of BBB cocultures using TNF-*α* and IFN-*γ*, alone or in combination, resulted in activation of endothelial cells at the molecular level, as evidenced by a strong upregulation of mRNA encoding the adhesion molecules ICAM-1 and VCAM-1 and a significant downregulation of transcripts encoding the tight junction proteins claudin-5, occludin, and zonula occludens-1 as well as the adhesion molecule ICAM-2 (Supplementary Table 2). These effects were most pronounced when TNF-*α* and IFN-*γ* were applied together, and TNF-*α* was found to be more potent than IFN-*γ* when applied separately. Also at the functional level, combined stimulation with TNF-*α* and IFN-*γ* (20.33 ± 1.05 Ωcm^2^) or with TNF-*α* alone (25.33 ± 0.89 Ωcm^2^) resulted in a significant decrease in TEER values as compared to untreated BBB cocultures (30.10 ± 0.78 Ωcm^2^, *p* < 0.001 and *p* < 0.01, respectively), while IFN-*γ* did not induce a significant decrease in TEER when applied separately (28.06 ± 0.08 Ωcm^2^, Supplementary Figure 1C). Paracellular permeability of the BBB cocultures was significantly increased following activation with both cytokines simultaneously, as evidenced by the higher recovery rate of the tracer molecule FITC dextran, after 2 and 3 hours. No statistically significant differences in paracellular permeability were found when TNF-*α* or IFN-*γ* were applied separately to the BBB coculture (Supplementary Figure 1D).

### 3.2. Proinflammatory Activation Significantly Upregulates Chemokine Secretion by Both hCMEC/D3 Endothelial Cells and Astrocytes

**Figure 1:**
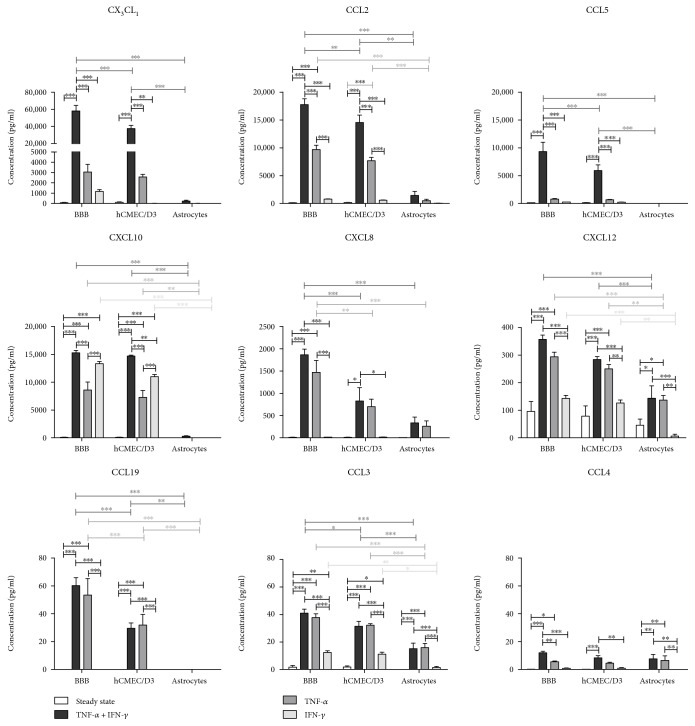
Chemokine secretion of hCMEC/D3 and astrocytes in mono- and coculture under steady-state conditions and after proinflammatory stimulation with TNF-*α* and/or IFN-*γ* (*n* = 4, ^∗^*p* < 0.05; ^∗∗^*p* < 0.01; ^∗∗∗^*p* < 0.001).

To analyze chemokine secretion by the different cellular components of the in vitro BBB model, supernatant of unstimulated and cytokine-activated hCMEC/D3 and astrocyte mono- and cocultures was analyzed by multiplex analysis (Figure 1). In steady-state conditions, low levels of CX_3_CL1, CXCL8, CXCL10, CCL2, CCL3, CXCL12, and CCL5 were detected in the supernatant of BBB cocultures. The production of these chemokines was mainly attributed to hCMEC/D3 endothelial cells; astrocytes were found to secrete only minute levels of CXCL12, CCL2, and CXCL8 and no CX_3_CL1, CXCL10, CCL5, and CCL3. No CCL4 and CCL19 could be detected in any of the conditions.

Activation with both TNF-*α* and IFN-*γ* resulted in a markedly increased secretion of all chemokines tested. The effect was particularly apparent for CCL5, CX_3_CL1, and CCL2, in which cases a 70-fold, 725-fold, and 160-fold increase in chemokine concentration was observed as compared to steady-state levels, respectively. Moreover, a synergistic effect of TNF-*α* and IFN-*γ* on chemokine secretion was seen. As in steady-state conditions, our results indicate that chemokine secretion by the hCMEC/D3 endothelial cells predominantly contributed to the chemokine levels detected in the BBB cocultures. Following activation with TNF-*α* alone, a strong upregulation of CXCL8, CXCL10, CCL2, CCL3, CCL4, and CXCL12 secretion was detected. CCL19 secretion was also induced by TNF-*α*, and its production could be entirely attributed to hCMEC/D3 endothelial cells since no CCL19 secretion could be detected by astrocytes. The secretion of CX_3_CL1 or CCL5 by endothelial cells nor astrocytes was affected after stimulation with TNF-*α*. In contrast, stimulation with IFN-*γ* alone was insufficient to significantly affect the secretion of the chemokines tested, except for secretion of CXCL10 and CCL3 by BBB cocultures and hCMEC/D3 but not astrocyte monocultures.

### 3.3. In Inflammatory Conditions, PBMC Penetrate the In Vitro BBB Model Less but Prominently Adhere to the Endothelial Layer

**Figure 2:**
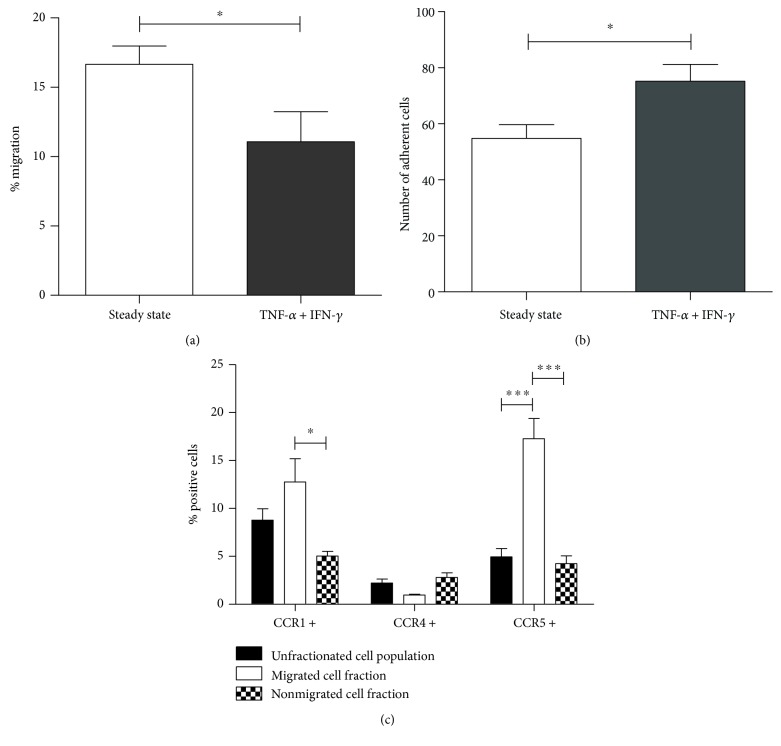
Migration of PBMC across the in vitro BBB model under steady-state and inflammatory conditions. Twenty hours following initiation of a migration assay, migrated and nonmigrated cell fractions were isolated for counting and phenotyping. (a) PBMC migrated more efficiently across steady-state BBB cocultures as compared to cytokine-activated BBB cocultures (*n* = 12). (b) After migration, a significantly higher number of CD45^+^ leukocytes was found to adhere to activated as compared to steady-state BBB cocultures (*n* = 3). (c) Compared to nonmigrated cells, cells that migrated through steady-state BBB cocultures expressed increased levels of CCR1 and CCR5. The percentage of CCR5-expressing cells was also higher in the migrated cell fraction than in the unfractionated population before migration. For CCR4 expression, no differences between the different cell fractions were observed (*n* = 4 for expression of CCR1 and CCR4 and *n* = 8 for expression of CCR5; ^∗^*p* < 0.05; ^∗∗^*p* < 0.01; ^∗∗∗^*p* < 0.001).

The marked chemokine secretion following proinflammatory stimulation of the BBB cocultures prompted us to investigate spontaneous migration of PBMC across steady-state and activated BBB cocultures. Notwithstanding the high chemokine levels produced by BBB cocultures following stimulation with TNF-*α* and IFN-*γ*, we found a significantly reduced proportion of PBMC crossing activated BBB cocultures as compared to steady-state BBB cocultures (11.07 ± 2.17% versus 16.66 ± 1.30%; *p* = 0.0381) (Figure 2(a)). However, we found an increased number of CD45^+^ cells adhering to activated cocultures as compared to steady-state BBB (*p* = 0.0143) (Figure 2(b)) using immunofluorescence imaging. Representative images are shown in Supplementary Figure 2.

In addition, we compared the expression levels of the chemokine receptors CCR1, CCR4, and CCR5 on cells that successfully crossed the in vitro BBB and the nonmigrated cell fraction by means of multiparametric flow cytometry. Interestingly, migrated cells displayed a significantly higher proportion of CCR1^+^ and CCR5^+^ cells (12.76 ± 2.44% and 17.28 ± 2.10%, respectively) as compared to nonmigrated cells (CCR1: 5.04 ± 0.49%, *p* < 0.01; CCR5: 4.24 ± 0.82%, *p* < 0.001) as well as to the unfractionated cell population before migration (CCR1: 8.79 ± 1.17%, *p* > 0.05; CCR5: 4.95 ± 0.88%, *p* < 0.001) in steady-state conditions (Figure 2(c)). Similar results were observed for the expression of CCR5 on migrated versus nonmigrated cells across a TNF-*α* and IFN-*γ*-stimulated in vitro BBB, whereas no significant difference in the proportion of CCR1^+^ cells between the migrated and nonmigrated cell population was observed (data not shown). No significant differences regarding the expression of CCR4 were detected in any of the conditions tested.

### 3.4. CCL3 Is Actively Transported across the In Vitro BBB Model Independent of Its Chemokine Receptor

**Figure 3:**
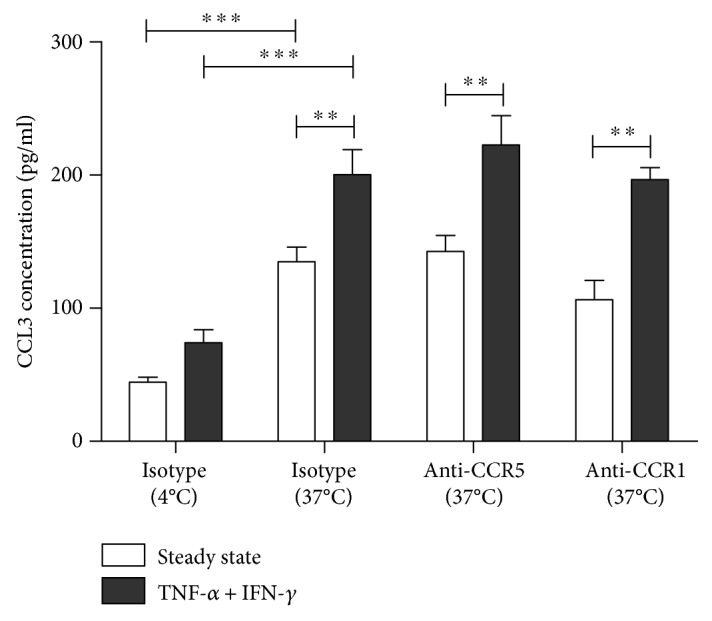
Basolateral-to-apical transport of exogenous CCL3 across the BBB in vitro is increased under inflammatory conditions but is not affected by preincubation of BBB cocultures with blocking antibodies targeting CCR1 or CCR5, suggesting that these chemokine receptors are not involved in the transendothelial transport of CCL3. To discriminate between active transendothelial transport, which takes place at 37°C only, and passive paracellular leakage of exogenously added CCL3 occurring at both 4°C and 37°C, experiments were performed at both 37°C and 4°C (*n* = 5, ^∗^*p* < 0.05; ^∗∗^*p* < 0.01; ^∗∗∗^*p* < 0.001).

Considering the higher proportion of CCR1- and CCR5-expressing cells in the migrated cell fraction, we next investigated whether the ligand of both chemokine receptors, CCL3, was transported from the abluminal side to the luminal side of the BBB. Noteworthy, expression of CCL3 is upregulated during neuroinflammation in the CNS, where it is mainly produced by cells located behind the BBB, including microglia, infiltrating immune cells, and astrocytes [15, 30, 31]. In the in vitro BBB model, levels of endogenously produced CCL3 over an assay period of 1 hour were below the ELISA's detection limit (i.e., <16 pg/ml) in both steady-state and inflammatory conditions (data not shown). We found that exogenous CCL3 added to the basolateral compartment is actively transported through the transcellular route, as evidenced by a significant increase in the CCL3 concentration detected in the medium of the apical compartment at 37°C as compared to 4°C (134.88 ± 11.08 pg/ml versus 44.26 ± 3.87 pg/ml; *p* < 0.01) (Figure 3). Both cell-mediated active transport and paracellular diffusion take place at a temperature of 37°C, whereas active transport is eliminated at 4°C, allowing only paracellular leakage of CCL3. Nevertheless, some paracellular leakage of CCL3 still takes place. Furthermore, active transendothelial transport of CCL3 further increased under inflammatory conditions, as evidenced by a significant increase in the concentration of CCL3 when BBB cocultures were stimulated with TNF-*α* and IFN-*γ* as compared to the steady-state condition (200.33 ± 18.86 pg/ml versus 134.88 ± 11.08 pg/ml; *p* < 0.001). No difference in paracellular permeability to CCL3 following proinflammatory activation was seen at 4°C. Importantly, blocking the chemokine receptors CCR1 or CCR5 did not affect transendothelial shuttling of CCL3 across the in vitro BBB model, in any of the conditions tested (Figure 3).

## 4. Discussion

Leukocyte trafficking to the immune-privileged CNS is tightly controlled in steady-state conditions, while dysregulation of this process and concomitant massive immune cell infiltration into the CNS are a hallmark of many neuroinflammatory disorders. Chemokines are critically involved in regulating leukocyte recruitment to the healthy as well as the inflamed CNS. As such, involvement of chemokines in the pathogenesis of several neuroinflammatory diseases has been demonstrated irrefutably [34, 35]. Understanding the mechanisms of leukocyte transmigration and the factors regulating this process is essential for the development of therapeutic strategies interfering with pathological immune cell infiltration, while leaving host immune surveillance mechanisms intact. Therefore, we developed an in vitro model of the BBB, consisting of a coculture of the cerebral microvascular endothelial cell line hCMEC/D3 and primary human astrocytes, to study chemokine secretion, chemokine transport, and leukocyte transmigration.

The in vitro BBB model was validated by assessing barrier function at the molecular and functional level. Once confluent, TEER values up to 38.20 Ωcm^2^ were recorded, in line with values reported by others using this cell line [36, 37]. Inflammatory conditions were mimicked by stimulation with TNF-*α* and/or IFN-*γ*, two cytokines known to activate endothelial cells [38] and to be critically involved in the pathogenesis of several neuroinflammatory diseases [39–45]. Activation of the BBB model with TNF-*α* and/or IFN-*γ* led to a strong reduction in the expression levels of mRNA encoding tight junction proteins, especially claudin-5 and occludin. In line with these findings, a significant decrease in TEER was measured after stimulation with TNF-*α* alone or together with IFN-*γ* but not with IFN-*γ* alone. In contrast, the expression of the adhesion molecules ICAM-1 and VCAM-1 was strongly upregulated after stimulation with TNF-*α* and IFN-*γ*, either alone or in combination, whereas ICAM-2 expression was significantly downregulated upon proinflammatory stimulation. These results are in agreement with the findings of Lopez-Ramirez and colleagues [46] and demonstrate a gene expression response that facilitates leukocyte adhesion and transmigration in neuroinflammation.

Analysis of chemokine secretion by the different cellular components of the in vitro BBB model revealed that hCMEC/D3 cells were the major source of chemokines under both steady-state and inflammatory conditions. All chemokines known to be constitutively expressed by primary hBMEC [7–12] could also be detected in low levels in the supernatant of hCMEC/D3 in mono- and cocultures, except for CCL4. Interestingly, CCL4 production was induced in hCMEC/D3 after TNF-*α* stimulation, while TNF-*α* apparently did not affect CCL4 expression by primary hBMEC [12]. Furthermore, our results indicate that TNF-*α* is far more potent in stimulating chemokine secretion than IFN-*γ*, except for CXCL10, which is also known as IFN-*γ*-inducible protein-10. Interestingly, TNF-*α* and IFN-*γ* have a strong synergistic effect on inducing the secretion of CCL2, CCL5, and CX_3_CL1. Similar effects have been described in endothelial cells of noncerebral origin [47]. Synergistic induction of chemokine secretion by proinflammatory cytokines provides a protective mechanism: it ensures amplification of the inflammatory response and at the same time eliminates the need for toxically high concentrations of proinflammatory cytokines to accumulate [48]. TNF-*α* and the combination TNF-*α*/IFN-*γ* are also potent stimulators of chemokine secretion by primary astrocytes, strongly inducing expression of CCL2, CCL3, CCL4, CCL5, CXCL8, CXCL9, CCL20, CXCL10, CXCL12, and CX_3_CL1 [31, 49–54]. In this study, we confirm upregulated expression of CCL3, CCL4, and CXCL12 upon activation with TNF-*α* or TNF-*α* and IFN-*γ* by human primary astrocytes. Although others demonstrated that astrocytes cultured in vitro are vigorous producers of chemokines, especially after proinflammatory stimulation [11, 12, 31, 49–52], they represented a minor source of chemokines in our in vitro BBB model. The culture conditions in our study, however, were optimized for endothelial cells and not astrocytes, which might have caused suboptimal performance of astrocytes. Moreover, the growth factor bFGF, which is a major constituent of the culture media used, was shown to inhibit activation of astrocytes after injury [55] and to reduce their secretion of proinflammatory cytokines such as IL-6 and TNF-*α* upon LPS stimulation [56]. It is plausible that medium-derived bFGF similarly affected chemokine secretion by astrocytes. Altogether, we demonstrate significantly upregulated chemokine secretion by both hCMEC/D3 endothelial cells and astrocytes following stimulation with proinflammatory signals.

Since our results confirmed that astrocytes are an important source of CCL3, we aimed to provide further insights into the mechanisms of CCL3 transport to the endothelial cell surface. We showed that neither CCR1 nor CCR5 participate in the trafficking of CCL3, despite active transport of this chemokine across the BBB in vitro suggesting that other mechanisms are involved in the active transport of CCL3 across the BBB in vitro. For instance, it has been shown that CCL3 binds to the atypical chemokine receptor Duffy antigen receptor for chemokines (DARC) [57], so DARC-mediated shuttling might be responsible for this. The fact that DARC expression [46, 58] but not CCR5 expression [59] is significantly upregulated upon activation of brain-derived endothelial cells with TNF-*α* and IFN-*γ* is in support of this hypothesis. This could also explain the increased active transendothelial flux of CCL3 under proinflammatory conditions observed in this study. Previously, Minten and colleagues [58] showed a similar role for the atypical chemokine receptor DARC in the transport of both CCL2 and CCL5 across TNF-*α*-activated murine brain endothelial cells.

Interestingly, CCL3 has been shown to be implicated in the trafficking of key effector cells to the brain during MS [14, 15] and its animal model experimental autoimmune encephalomyelitis [16–18], Alzheimer's disease [19, 20], seizure disorders [21–25], and viral [26, 27] as well as bacterial neuroinflammation [28, 29]. Here, we found a significantly elevated proportion of CCR1^+^ and CCR5^+^ cells in the migrated fraction as compared to nonmigrating cells or to the unfractionated cell population before migration. Accordingly, others demonstrated enrichment of CCR1- and CCR5-expressing cells in the demyelinating lesions and cerebrospinal fluid of MS patients [15, 60]. However, in our hands, the overall migration of PBMC was significantly lower across activated BBB cocultures as compared to steady-state BBB cocultures, despite the elevated expression of mRNA encoding adhesion molecules, the downregulation of mRNA encoding tight junction molecules, and the pronounced induction of chemokine secretion under inflammatory conditions, including CCL3, by the BBB endothelial cells upon proinflammatory stimulation. It is likely that changes occurring in the activated endothelial cells have promoted adhesion rather than transmigration of the PBMC. Indeed, fluorescence microscopy imaging revealed that significantly more CD45^+^ white blood cells adhered to the CD31^+^ endothelial cells in inflamed as compared to steady-state BBB cocultures, which is in agreement with previous reports [61]. Alternatively, it has been reported that stimulation of endothelial cells with TNF-*α* and IFN-*γ* causes the adhesion molecule CD31 or platelet endothelial cell adhesion molecule-1 (PECAM-1) to disappear from interendothelial cell junctions, which correlates with a marked decrease in transmigration of leukocytes through endothelial cell monolayers [62]. Similarly, we found highly disorganized PECAM-1 expression by hCMEC/D3 endothelial cells in BBB cocultures after activation with TNF-*α* and IFN-*γ* (Supplementary Figure 2B).

In conclusion, we have demonstrated a paradoxical decrease in PBMC transmigration after BBB activation, despite increased secretion of chemokines by the BBB proper as well as active transport of CNS-derived CCL3 to the vascular lumeninal side. This suggests that additional pathways originating from beyond the BBB are involved in the increased immune cell infiltration seen during neuroinflammation in vivo, as such highlighting the complex nature of the mechanisms underlying immune cell infiltration into the immune-specialized compartment of the CNS as well as the pivotal role of the BBB in this process. Nonetheless, we have shown, for the first time, active transport of CCL3 across the human BBB in vitro and further characterized the chemokine secretion pattern of the cells comprising the BBB. Targeting chemokines and chemokine receptors has been proposed in the treatment of neuroinflammation. Although to date clinical application of this kind of therapies is limited, the first studies show promising results [63].

## Supplementary Material

Supplementary table 1. Validation data of gene-specific primers. Assay efficiency was determined using a seven-point standard curve from 20 copies to 20 million copies. Ideally, the efficiency equals 100%, representing a perfect doubling of template at every cycle. Typically, good assay efficiencies range between 90-110%. R2 represents the linearity of the standard curve and how well the standard curve data points fit the linear regression line. Acceptable values are > 0.98. cDNA Cq is the Cq value obtained from 25 ng of cDNA transcribed from universal RNA when performing wet-lab validation of the assay. cDNA Tm is the melting temperature of the amplicon when running a melt curve analysis. gDNA Cq is the Cq value obtained when running the assay with 2.5 ng of genomic DNA, a more than moderate level of genomic DNA contamination. The specificity is represented by the percentage of specific amplicon reads as measured by next generation sequencing (NGS). While 100% specificity is desirable, small decreases in specificity (<1%) can be due to NGS read errors. Information obtained from Bio-Rad validation reports delivered with gene-specific primers. Supplementary table 2. Proinflammatory stimulation of the in vitro BBB model activates hCMEC/D3 endothelial cells with altered expression of adhesion markers and tight junction proteins. When BBB co-cultures were treated with the proinflammatory cytokines TNF-α and IFN-γ alone or in combination, endothelial cells were activated on the molecular level, as evidenced by a strong upregulation of adhesion molecule mRNA expression and significant downregulation of transcripts encoding tight junction proteins. mRNA encoding ICAM-1 and VCAM-1 showed a significant upregulation upon activation of BBB cultures stimulated with IFN-γ and an even stronger upregulation after stimulation with TNF-α, while the 2 cytokines combined led to the highest level of both ICAM-1 and VCAM-1 mRNA expression. No difference was found for L1CAM mRNA expression levels. ICAM-2 expression was significantly downregulated upon treatment with TNF-α and IFN-γ, albeit no change in its expression was found upon treatment with each of the proinflammatory cytokines separately. Following treatment of the BBB with IFN-γ and TNF-α combined, mRNA expression levels of the tight junction molecules occludin, TJP-1 and claudin were significantly decreased. Although less pronounced, the cytokines separately also induced a marked reduction in the expression levels of mRNA encoding tight junction proteins. Results are expressed as fold regulation compared to steady-state BBB co-cultures (n=3, ∗∗ p<0.01). Supplementary Figure 1. Validation of the in vitro blood-brain barrier (BBB) model and its activation by proinflammatory cytokines. (A) Transendothelial electrical resistance (TEER) of the in vitro BBB model was measured at several time points during the culture period. TEER values gradually increased over time. TEER values measured from day 10 on were significantly higher than the initial value determined on day 3. Accordingly, subsequent functional assays were performed between day 10 and 13 after initiation of the co-culture (n=6). (B) RT-qPCR analysis of the gene expression profile of hCMEC/D3 co-cultured with astrocytes as compared to hCMEC/D3 mono-cultures reveals a limited impact of astrocyte co-culturing. Of the selected markers, only mRNA encoding the tight junction protein occludin was found to be significantly upregulated in hCMEC/D3-astrocyte co-cultures as compared to hCMEC/D3 in mono-culture (n=3, ∗∗ p<0.01). (C) Measurements of TEER were performed to analyze the effects of astrocyte co-culturing and proinflammatory stimulation on hCMEC/D3 endothelial cell barrier function. TEER values of BBB co-cultures were not significantly higher when compared to those of hCMEC/D3 mono-cultures (n=9). Activation of BBB co-cultures with TNF-α or TNF-α in combination with IFN-γ, but not with IFN-γ alone, induces a significant reduction in TEER (n=17). (D) Stimulation of BBB co-cultures with TNF-α + IFN-γ, but not with either of the cytokines separately, induces a significant increase in permeability to the tracer molecule FITC-dextran, another measure for barrier function (n=5, ∗ p<0.5; ∗∗ p<0.01; ∗∗∗ p<0.001). Supplementary Figure 2. Representative images of immunofluorescence analysis of the adherence by CD45+ PBMC to CD31+ endothelial cells of steady-state and cytokine-activated BBB co-cultures, after transmigration assay. Transmigration assays were performed as described in the Material and Methods section. After harvesting, BBB co-cultures were fixated in 4% paraformaldehyde. Using indirect immunofluorescence, the adherence of CD45+ cells (FITC, green) to the CD31+ hCMEC/D3 endothelial cells (Cy3, red) in both steady-state (A) and inflamed (B) BBB co-cultures was studied. Remarkably, hCMEC/D3 endothelial cells in cytokine-activated BBB co-cultures displayed highly disorganized CD31 expression.
