# Supplementary material for: Increased Transendothelial Transport of CCL3 Is Insufficient to Drive Immune Cell Transmigration through the Blood–Brain Barrier under Inflammatory Conditions In Vitro
Source: Mediators Inflamm. 2017 May 25;2017:6752756. doi: 10.1155/2017/6752756 (PMC5463143; doi:10.1155/2017/6752756)

SUPPLEMENTARY TABLE LEGENDS

Supplementary table 1. Validation data of gene-specific primers

Assay efficiency was determined using a seven-point standard curve from 20 copies to 20 million copies. Ideally, the efficiency equals 100%, representing a perfect doubling of template at every cycle. Typically, good assay efficiencies range between 90-110%. R^2 represents the linearity of the standard curve and how well the standard curve data points fit the linear regression line. Acceptable values are > 0.98. cDNA Cq is the Cq value obtained from 25 ng of cDNA transcribed from universal RNA when performing wet-lab validation of the assay. cDNA Tm is the melting temperature of the amplicon when running a melt curve analysis. gDNA Cq is the Cq value obtained when running the assay with 2.5 ng of genomic DNA, a more than moderate level of genomic DNA contamination. The specificity is represented by the percentage of specific amplicon reads as measured by next generation sequencing (NGS). While 100% specificity is desirable, small decreases in specificity (<1%) can be due to NGS read errors. *Information obtained from Bio-Rad validation reports delivered with gene-specific primers.*

Supplementary table 2. Proinflammatory stimulation of the *in vitro* BBB model activates hCMEC/D3 endothelial cells with altered expression of adhesion markers and tight junction proteins

When BBB co-cultures were treated with the proinflammatory cytokines TNF-α and IFN-γ alone or in combination, endothelial cells were activated on the molecular level, as evidenced by a strong upregulation of adhesion molecule mRNA expression and significant downregulation of transcripts encoding tight junction proteins. mRNA encoding ICAM-1 and VCAM-1 showed a significant upregulation upon activation of BBB cultures stimulated with

IFN-γ and an even stronger upregulation after stimulation with TNF-α, while the 2 cytokines combined led to the highest level of both ICAM-1 and VCAM-1 mRNA expression. No difference was found for L1CAM mRNA expression levels. ICAM-2 expression was significantly downregulated upon treatment with TNF-α and IFN-γ, albeit no change in its expression was found upon treatment with each of the proinflammatory cytokines separately. Following treatment of the BBB with IFN-γ and TNF-α combined, mRNA expression levels of the tight junction molecules occludin, TJP-1 and claudin were significantly decreased. Although less pronounced, the cytokines separately also induced a marked reduction in the expression levels of mRNA encoding tight junction proteins. Results are expressed as fold regulation compared to steady-state BBB co-cultures (n=3, ** $p<0.01$).

SUPPLEMENTRARY FIGURE LEGENDS

Supplementary Figure 1. Validation of the *in vitro* blood-brain barrier (BBB) model and its activation by proinflammatory cytokines. (A) Transendothelial electrical resistance (TEER) of the *in vitro* BBB model was measured at several time points during the culture period. TEER values gradually increased over time. TEER values measured from day 10 on were significantly higher than the initial value determined on day 3. Accordingly, subsequent functional assays were performed between day 10 and 13 after initiation of the co-culture (n=6). **(B)** RT-qPCR analysis of the gene expression profile of hCMEC/D3 co-cultured with astrocytes as compared to hCMEC/D3 mono-cultures reveals a limited impact of astrocyte co-culturing. Of the selected markers, only mRNA encoding the tight junction protein occludin was found to be significantly upregulated in hCMEC/D3-astrocyte co-cultures as compared to hCMEC/D3 in mono-culture (n=3, ** $p<0.01$). **(C)** Measurements of TEER were performed to analyze the effects of astrocyte co-culturing and proinflammatory stimulation on hCMEC/D3 endothelial cell barrier function. TEER values of BBB co-cultures were not significantly higher when compared to those of hCMEC/D3 mono-cultures (n=9). Activation of BBB co-cultures with TNF-α or TNF-α in combination with IFN-γ, but not with IFN-γ alone, induces a significant reduction in TEER (n=17). **(D)** Stimulation of BBB co-cultures with TNF-α + IFN-γ, but not with either of the cytokines separately, induces a significant increase in permeability to the tracer molecule FITC-dextran, another measure for barrier function (n=5, * $p<0.5$; ** $p<0.01$; *** $p<0.001$).

Supplementary Figure 2. Representative images of immunofluorescence analysis of the adherence by CD45$^+$ PBMC to CD31$^+$ endothelial cells of steady-state and cytokine-activated BBB co-cultures, after transmigration assay. Transmigration assays were performed as described in the Material and Methods section. After harvesting, BBB co-cultures were fixated in 4% paraformaldehyde. Using indirect immunofluorescence, the adherence of CD45$^+$ cells (FITC, green) to the CD31$^+$ hCMEC/D3 endothelial cells (Cy3, red) in both steady-state (A) and inflamed (B) BBB co-cultures was studied. Remarkably, hCMEC/D3 endothelial cells in cytokine-activated BBB co-cultures displayed highly disorganized CD31 expression.

SUPPLEMENTRARY TABLES

Supplementary table 1.

Gene symbol	Gene name	Protein name	Assay design	Efficiency (%)	R^2	cDNA Cq	cDNA Tm (°C)	gDNA Cq	Specificity (%)
**CLDN5**	Claudin 5	CLDN-5	Exonic	102	0.9993	28.07	90.5	24.16	100
**OCLN**	Occludin	OCLN	Exonic	90	0.9996	22.47	78	23.75	99.06
**TJP1**	Tight junction protein 1 (zona occludens 1)	TJP-1, ZO-1	Intron-spanning	94	0.999	20.3	85	/	100
**SLC2A1**	Solute carrier family 2, member 1 (glucose transporter 1)	SLC2A-1, GLUT-1	Intron-spanning	106	0.9976	19.57	89.5	36.66	100
**ICAM1**	Intercellular adhesion molecule 1	ICAM-1	Exonic	99	0.9985	19.49	82.5	23.54	100
**ICAM2**	Intercellular adhesion molecule 2	ICAM-2	Exonic	98	0.9993	22.34	86.5	22.98	100
**L1CAM**	L1 cell adhesion molecule	L1CAM	Exonic	94	0.9995	21.71	82.5	23.92	100
**VCAM1**	Vascular adhesion molecule 1	VCAM1	Intron-spanning	102	0.9991	22.79	79.5	34.81	100
**ACTB**	Actin, beta	ACTB	Exonic	103	0.9939	15.155	82	22.445	/

Supplementary table 2.

	TNF-α	IFN-γ	TNF-α + IFN-γ
**CLDN-5**	-2,23**	-2,78**	-3,26**
**ICAM-1**	13,25**	3,43**	13.62**
**ICAM-2**	-2,15	-1,04	-2,28**
**L1CAM**	1,32	1,95	1,15
**OCLN**	-2,26**	-2,06**	-5,33**
**SLC2A-1**	-1,67**	-1,45	-2,50**
**TJP-1**	-1,40**	-1,06	-1,97**
**VCAM-1**	288,29**	12,24**	555,37**

SUPPLEMENTARY FIGURE 1A

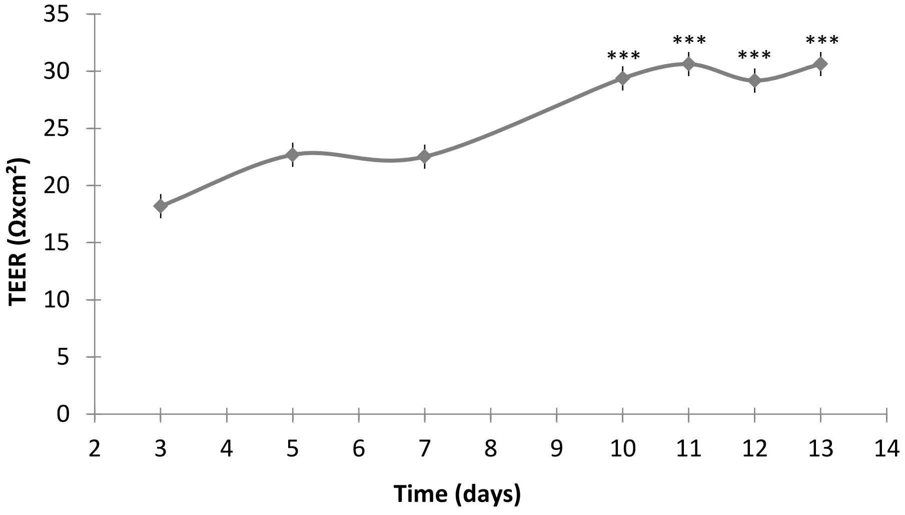

SUPPLEMENTARY FIGURE 1B

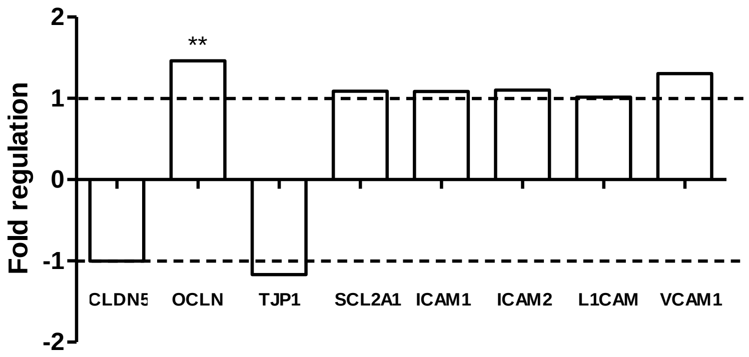

SUPPLEMENTARY FIGURE 1C

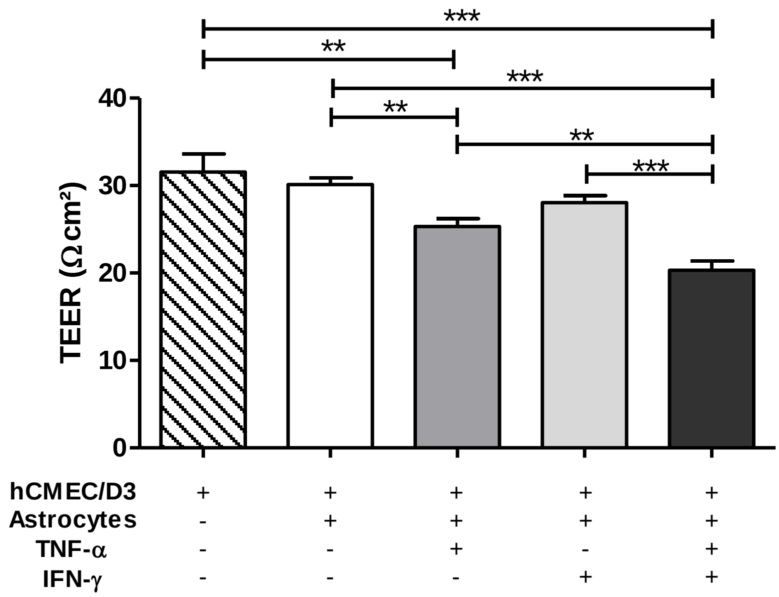

SUPPLEMENTARY FIGURE 1D

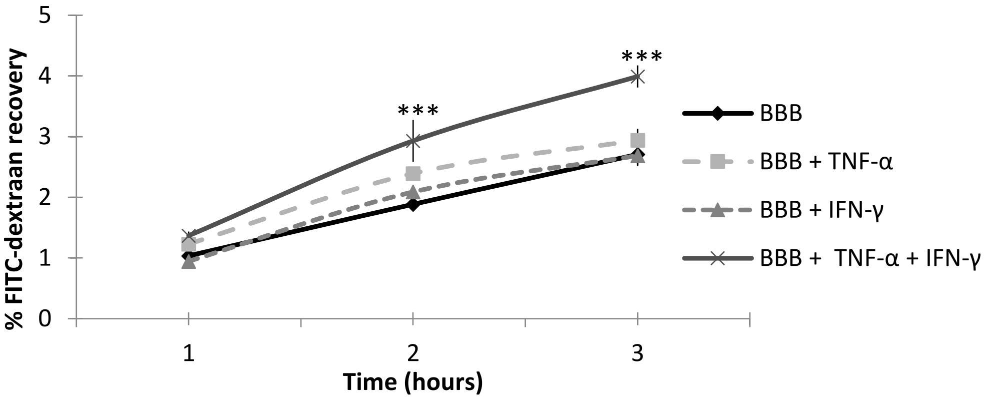

SUPPLEMENTARY FIGURE 2A

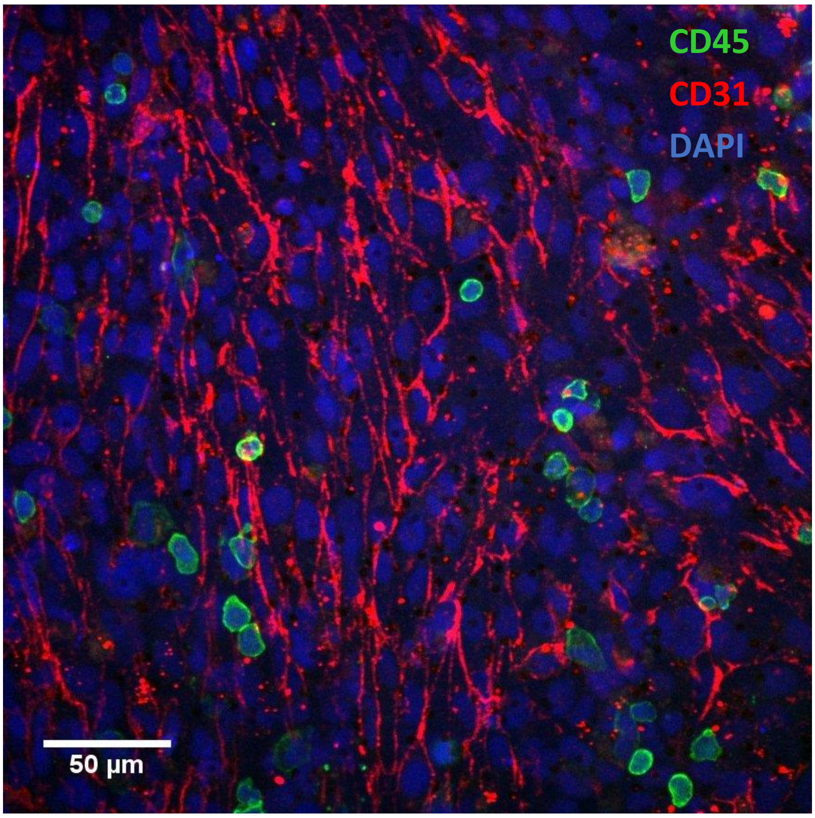

SUPPLEMENTARY FIGURE 2B

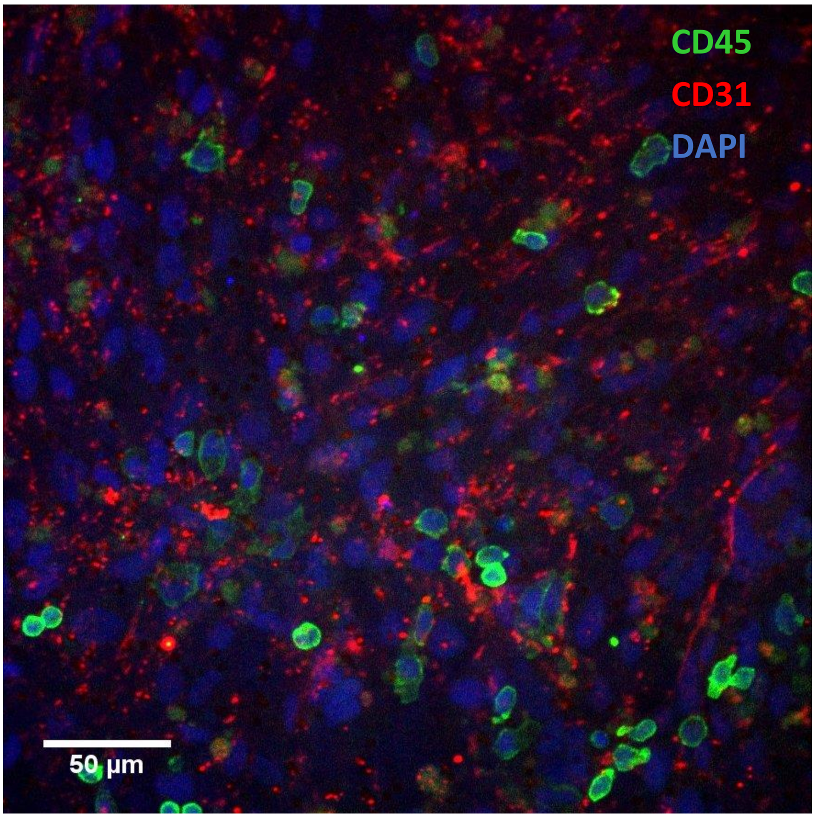

Supplement: Supplementary file 1 — Supplementary table 1. Validation data of gene-specific primers. Assay efficiency was determined using a seven-point standard curve from 20 copies to 20 million copies. Ideally, the efficiency equals 100%, representing a perfect doubling of template at every cycle. Typically, good assay efficiencies range between 90-110%. R2 represents the linearity of the standard curve and how well the standard curve data points fit the linear regression line. Acceptable values are > 0.98. cDNA Cq is the Cq value obtained from 25 ng of cDNA transcribed from universal RNA when performing wet-lab validation of the assay. cDNA Tm is the melting temperature of the amplicon when running a melt curve analysis. gDNA Cq is the Cq value obtained when running the assay with 2.5 ng of genomic DNA, a more than moderate level of genomic DNA contamination. The specificity is represented by the percentage of specific amplicon reads as measured by next generation sequencing (NGS). While 100% specificity is desirable, small decreases in specificity (<1%) can be due to NGS read errors. Information obtained from Bio-Rad validation reports delivered with gene-specific primers. Supplementary table 2. Proinflammatory stimulation of the in vitro BBB model activates hCMEC/D3 endothelial cells with altered expression of adhesion markers and tight junction proteins. When BBB co-cultures were treated with the proinflammatory cytokines TNF-α and IFN-γ alone or in combination, endothelial cells were activated on the molecular level, as evidenced by a strong upregulation of adhesion molecule mRNA expression and significant downregulation of transcripts encoding tight junction proteins. mRNA encoding ICAM-1 and VCAM-1 showed a significant upregulation upon activation of BBB cultures stimulated with IFN-γ and an even stronger upregulation after stimulation with TNF-α, while the 2 cytokines combined led to the highest level of both ICAM-1 and VCAM-1 mRNA expression. No difference was found for L1CAM mRNA expressi [file 6752756.f1.pdf]
