# Supplementary figures and images for: Increased Transendothelial Transport of CCL3 Is Insufficient to Drive Immune Cell Transmigration through the Blood–Brain Barrier under Inflammatory Conditions In Vitro
Source: Mediators Inflamm. 2017 May 25;2017:6752756. doi: 10.1155/2017/6752756 (PMC5463143; doi:10.1155/2017/6752756)

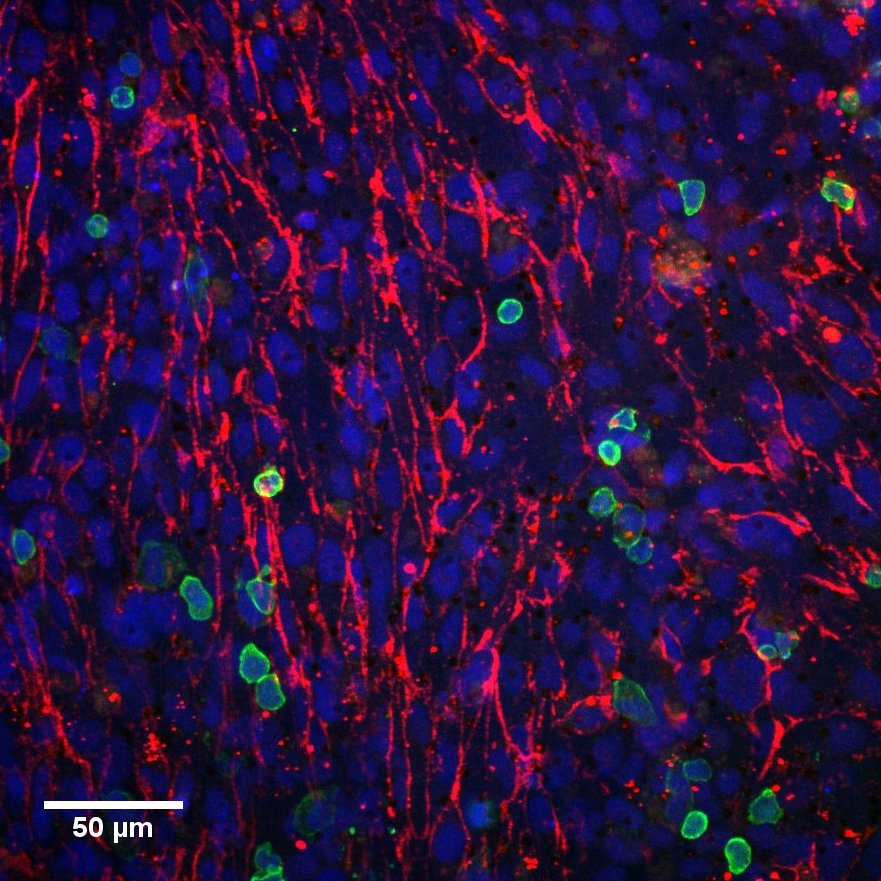


**CD45**

**CD31**

**DAPI**

Supplement: Supplementary file 7 [file 6752756.f7.doc]

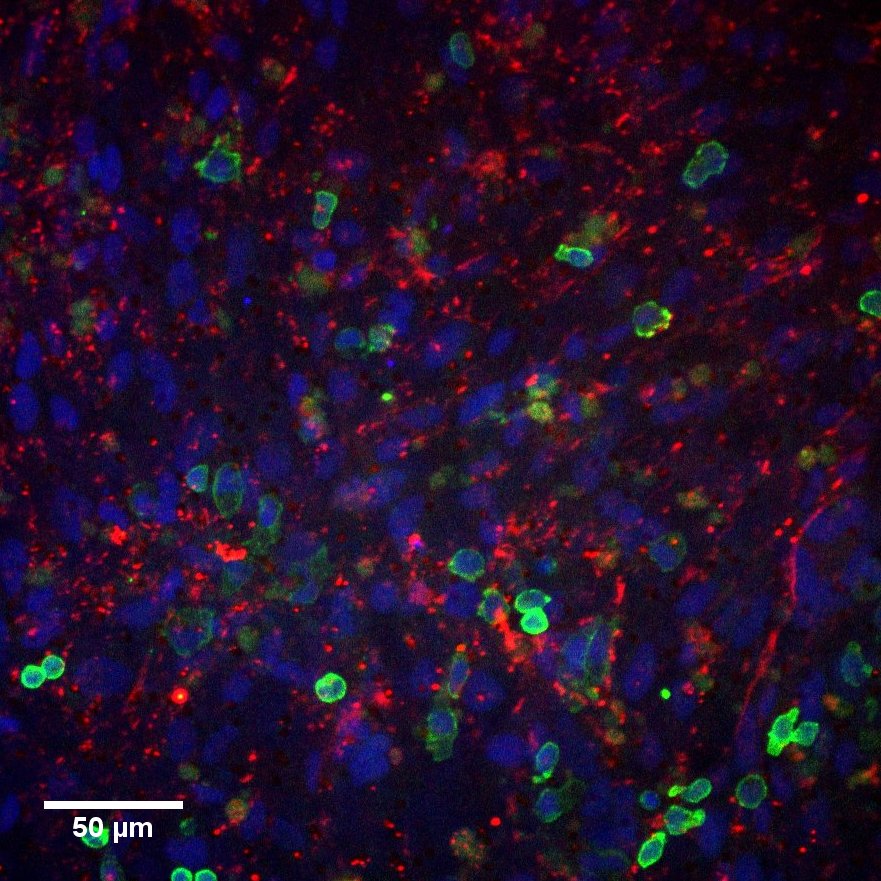


**CD45**

**CD31**

**DAPI**

Supplement: Supplementary file 8 [file 6752756.f8.doc]
